# miR-16-5p, miR-103-3p, and miR-27b-3p as Early Peripheral Biomarkers of Fetal Growth Restriction

**DOI:** 10.3389/fped.2021.611112

**Published:** 2021-03-11

**Authors:** Salvatore Tagliaferri, Pasquale Cepparulo, Antonio Vinciguerra, Marta Campanile, Giuseppina Esposito, Giuseppe Maria Maruotti, Fulvio Zullo, Lucio Annunziato, Giuseppe Pignataro

**Affiliations:** ^1^Division of Obstetrics and Gynecology, Department of Neuroscience, Reproductive and Dentistry Sciences, School of Medicine, University of Naples “Federico II”, Naples, Italy; ^2^Division of Pharmacology, Department of Neuroscience, Reproductive and Dentistry Sciences, School of Medicine, University of Naples “Federico II”, Naples, Italy; ^3^IRCCS SDN Napoli, Naples, Italy

**Keywords:** biomarkers, fetal growth restriction, hypoxia, microRNA, fetal growth restriction, FGR

## Abstract

Current tests available to diagnose fetal hypoxia *in-utero* lack sensitivity thus failing to identify many fetuses at risk. Emerging evidence suggests that microRNAs derived from the placenta circulate in the maternal blood during pregnancy and may be used as non-invasive biomarkers for pregnancy complications. With the intent to identify putative markers of fetal growth restriction (FGR) and new therapeutic druggable targets, we examined, in maternal blood samples, the expression of a group of microRNAs, known to be regulated by hypoxia. The expression of microRNAs was evaluated in maternal plasma samples collected from (1) women carrying a preterm FGR fetus (FGR group) or (2) women with an appropriately grown fetus matched at the same gestational age (Control group). To discriminate between early- and late-onset FGR, the study population was divided into two subgroups according to the gestational age at delivery. Four microRNAs were identified as possible candidates for the diagnosis of FGR: miR-16-5p, miR-103-3p, miR-107-3p, and miR-27b-3p. All four selected miRNAs, measured by RT-PCR, resulted upregulated in FGR blood samples before the 32nd week of gestation. By contrast, miRNA103-3p and miRNA107-3p, analyzed between the 32nd and 37th week of gestation, showed lower expression in the FGR group compared to aged matched controls. Our results showed that measurement of miRNAs in maternal blood may form the basis for a future diagnostic test to determine the degree of fetal hypoxia in FGR, thus allowing the start of appropriate therapeutic interventions to alleviate the burden of this disease.

## Introduction

Hypoxia may occur acutely during labor and birth, or develop gradually across pregnancy in cases of fetal growth restriction (FGR) due to placental dysfunction (1, 2). According to the time of growth restriction onset we distinguish the early-onset from the late-onset FGR. The early-onset FGR, occurring at <32 weeks of pregnancy, represents 20–30% of all FGRs and it is associated with severe placental insufficiency, Doppler abnormalities, and preeclampsia (50% of cases). The pathophysiology of early-onset is a reduction of more than 30% in the vascular area of the chorionic villi, resulting in severe placental insufficiency and chronic fetal hypoxia (3–5).

The late-onset FGR, from 32nd to 37th weeks of gestation, represent the 70–80% of FGR cases, it is frequently associated with mild placental insufficiency and normal Doppler velocimetry. The pathophysiology of the late-onset is the insufficient maturation of the chorionic villi or the reduction of their area in the placenta (4).

Fetal growth restriction, also known as intrauterine growth restriction (IUGR), was firstly erroneously defined as a condition characterized as failure of the fetus to achieve his/her genetically determined growth potential, with birth weight less than the 10th percentile (5). More recently, FGR has been better defined as a pathologic condition for a fetus that has not attained its biologically determined growth potential, for that particular gestational age and the definition was correlated to reduction of abdominal circumference, fetal weight estimation and Doppler changes (6).

FGR is estimated to be ~5–8% in the general obstetric population, frequently the etiology is the placental dysfunction (1). FGR is related to an increased risk of perinatal complications and important long term implications for the infant neurodevelopment (2).

There are no effective intrauterine therapeutic options for FGR, and management relies on early detection and timely delivery before stillbirth occurs (7). Therefore, clinicians utilize a combination of tests to assess fetal well-being to determine whether the fetus is at risk of succumbing to hypoxia. These include ultrasound assessment of fetal growth, Doppler velocimetry of fetal vessels, electronic Fetal Heart Rate (FHR) monitoring, and fetal movement counting (8). However, there is no clear evidence that antenatal cardiotocography (CTG) improves perinatal outcome (9, 10). Therefore, the development of an alternative assessment for fetal hypoxia is greatly needed.

In recent years, circulating placental specific microRNAs (miRNAs) have been identified in the maternal blood during pregnancy, which may reflect both physiological and pathological placental conditions (11). This offers a potential new avenue to develop a screening test for pregnancy complications. In fact, miRNAs are 19–25 nucleotide non-coding RNAs that play important roles in the regulation of a wide variety of genes by inhibiting mRNA translation or cleaving mRNA. Hypoxia has a profound effect on gene regulation and miRNAs play a vital role in the cellular response to hypoxia (12).

In the present work, we chose to investigate the expression of nine microRNAs whose expression is known to be affected by hypoxic stimulus. In fact, miR-103-3p,−107-3p, and−16-5p are members of miR-15/107 family, whose brain levels are induced by ischemic stroke, and their inhibition by antagomir has been considered as a novel therapeutic approach for brain ischemia (13, 14). MiR-27b-3p and miR-223-5p are both involved in the regulations of genes whose expressions are important for the pathogenesis of stroke damage (15, 16). Circulating miR-218a-5p was reported as prominent plasma biomarker for risk of acute ischemic stroke (17), whereas miR-101a-3p levels correlated with disease severity and NIHSS score (18). Furthermore, in our recent study, prevention of cerebral let-7a and miR-143 overexpression induced by ischemic stroke was proposed as new potential strategy in stroke intervention (19).

Therefore, in the present study, we hypothesized that measuring these hypoxia-regulated circulating miRNAs in the maternal blood could represent a promising strategy to identify biomarkers for *in-utero* fetal hypoxia.

## Materials and Methods

### Experimental Groups

All subjects were pregnant Caucasian women between 18 and 46 years old hospitalized at the Department of Obstetrical-Gynecological and Urological Science and Reproductive Medicine of the Federico II University (Italy) from January to August 2019. Written informed consent was obtained prior to inclusion in the study protocol.

The FGR group was defined by estimated weight below the 10th centile (1) and/or estimated abdominal circumference below the 10th centile, combined with UA-PI >95th centile in early-onset FGR and with CPR <5th centile or UA-PI >95th centile in late-onset FGR.

To discriminate between early- and late-onset FGR, the study population was divided into two subgroups according to the gestational age at delivery: (group I) <32nd weeks of gestation; (group II) from 32nd to 37th weeks of gestation (6).

Healthy group included fetuses whose growth was appropriate for gestational age without any chromosomal or major congenital anomalies. Healthy pregnancies were admitted in hospital for preterm contractions, without premature rupture of membranes (PPROM) or vaginal swab positive for infections, resolved spontaneously without premature delivery, or maternal renal colic resolved spontaneously after few days. All healthy pregnant women subsequently delivered appropriately grown neonates at term without obstetric complications. Healthy group was divided into two subgroups to match them to FGR fetuses: (group I) <32nd weeks of gestation; (group II) from 32nd to 37th weeks of gestation.

Starting from a population of 1400 pregnant women, 77 pregnant women composed of 34 complicated by FGR and 43 with healthy fetuses fulfilled the criteria of the study.

### Inclusion Criteria

Inclusion criteria were Caucasian ethnic; singleton pregnancy; certain pregnancy dating (calculated from the first day of the last menstrual period and confirmed by ultrasound measurements, according to the population nomograms) (20); gestational age from 26th week; cCTGs with a signal loss of <15% over the whole record; cCTG monitoring was recorded once a week for FGR and Healthy fetuses; delivery indication only for fetal condition in FGR group. Newborn baby data (sex, weight, Apgar score, malformation at birth, umbilical artery gas and pH values, access to neonatal intensive care) were collected. Newborns with missing data or inadequate umbilical cord samples at birth (insufficient blood sampling and/or errors in pH and gas analysis by the pH meter) were excluded.

Maternal whole blood samples were collected from women carrying a preterm FGR fetus (*n* = 34) and gestation matched controls (*n* = 43).

We excluded pre-existing maternal disease, such as hypertension, diabetes, epilepsy, heart and renal disease, lung disease, autoimmune disorders, malnutrition, and severe anemia. Maternal diseases arising or related to pregnancy were also excluded. Smoking, coffee, alcohol, and drug abuse were excluded. Fetuses with chromosomal, genetic, and congenital anomalies diagnosed during pregnancy or after birth were excluded.

Therefore, only FGR due to impaired uteroplacental blood flow were included. The term “placental insufficiency” used in the study refers to the most important pregnancy complication in which the placenta does not function adequately, because of insufficient invasion and remodeling of maternal spiral arteries that then leads to downstream effects resulting from reduced perfusion as FGR. To capture placental dysfunction across the phenotype of early- and late-onset FGR, inclusion of UA, MCA, and Ductus venosus Doppler values were required.

As regards delivery, only pregnant women whose elective or urgent delivery occurred for fetal indication were considered in the FGR group, excluding maternal or gestational indications. In this way, they were selected only pregnancies complicated by fetal growth retardation, avoiding the overlap with preeclampsia and gestational diabetes that share common placental vascular alterations due to angiogenic disbalance.

### Assessment of Growth Restriction Severity

The severity of the growth restriction was assessed by ultrasound biometry, Doppler velocimetry of umbilical artery (UA), middle cerebral artery (MCA), ductus venosus (DV), and cCTG. PI of UA and DV was considered abnormal when it was >95th centile for gestational age (3); when absent or reverse end-diastolic flow in DV (2, 21) and in UA were detected or MCA PI was <5th centile (22, 23).

The tests were made with the same frequency in all cases.

### Plasma Samples Collection

Human blood samples were withdrawn from pregnant women and collected in BD Vacutainer tubes (K3 EDTA 5.4 mg). For plasma separation, blood samples were centrifuged in ALC PK 120 centrifuge at 1,500 × g (2,900 rpm) for 8 min at room temperature. The supernatant plasma was transferred to sterile 2.0 ml tubes and centrifuged in Eppendorf centrifuge at 11,000 rpm to clean the sample from any cellular residues. The absorbance at 415 nm of a 50 μl aliquot for each sample was measured in a Bio-rad Microplate Reader to evaluate the possibility of a previous haemolytic process during blood withdrawal. Only samples with absorbance values <0.5OD were used for next analyses.

Maternal plasma samples were collected after 2 weeks of hospitalization and at least 48 h before delivery in all cases. In this way, pregnant women shared the same hospital setting and they had well-understood and accepted intensive fetal surveillance until delivery. It was collected a single maternal plasma sample per patient.

### MicroRNA Isolation and Assessment by Real-Time Polymerase Chain Reaction

MiRNA isolation from plasma samples was performed by using miRNeasy Serum/Plasma Kit (Qiagen) according to the manufacturer's protocol. Precise volumes (5 μl) of RNA were retrotranscribed by using High Capacity cDNA Reverse Transcription Kit (Applied Biosystems) and Taqman probes (Taqman MicroRNA Assays, Thermo Fisher Scientific), following TaqMan Small RNA Assays Protocol (16°C for 30 min, 42°C for 30 min, and 85°C for 5 min). Quantitative real-time polymerase chain reaction was performed with TaqMan Universal PCR Master Mix II (Applied Biosystems) in a 7500 Fast Real-Time PCR System (AB Applied Biosystems). cDNA samples were amplified simultaneously in triplicate in 1 assay run, following the protocol for Taqman assays: 50°C for 2 min, 95°C for 10 min, 40 cycles of amplification of 95°C for 15 s, and 60°C for 1 min. Results were analyzed and exported with 7500 Fast System SDS Software.

Values were expressed as means ± SEM. In particular, real-Time PCR results are expressed as fold change (2^−ΔΔCt^) compared to the control group, following the instructions provided by the literature (24). Briefly, difference between Ct values of gene of interest and internal control (ΔCt) is calculated for both control sample and sample of interest. Then, difference between ΔCt of sample of interest and control sample (ΔΔCt) is calculated. Fold change of gene expression of samples of interest compared to control sample is calculated as 2^−ΔΔCt^.

TaqMan probes used are the following: hsa-miR-16-5p (ID: 000391); hsa-miR-103-3p (ID: 000439); hsa-miR-107 (ID: 000443); hsa-miR-27b-3p(ID: 000409); miRNA Control Assay U6 snRNA (ID: 001973).

### Information on microRNA-Gene-Disease Ontology Interactions

In order to provide a list of predicted targets for the four selected miRNA, whose expression in blood samples of pregnant women was affected in case of FGR, we firstly interrogated miRWalk (available: http://mirwalk.umm.uni-heidelberg.de), MiRDB (available: http://mirdb.org/miRDB/) and Targetscan databases (available: http://www.targetscan.org/vert_72/). Then we confirmed our results with the most recent database miRPathDB v2.0 (https://mpd.bioinf.uni-sb.de/overview.html). These databases include predicted and validated biological targets of miRNAs, by searching for the presence of conserved sites that match the seed region of each miRNA, to provide information on putative interaction.

### Statistical Analysis

Statistical analysis was performed with GraphPad Prism 5.0 (GraphPad Software, Inc., San Diego, CA), using *t*-test for groups unpaired. Statistical significance was accepted at the 95% confidence level (*p* < 0.05).

## Results

### No Significant Differences Between Healthy and FGR Fetuses Were Detected Using Computerized FHR Parameters

All pregnant women were hospitalized at the Division of Obstetrics and Gynecology, School of Medicine, University of Naples “Federico II”, so that each group and subgroup included patients with the same lifestyle, that is the same low-calorie diet with similar energy consumption, as well as no alcohol or smoking. Moreover, sharing the same clinical environment for long time helps pregnant women with FGR to better understand and accept the intensive pregnancy monitoring, to trust medical choices and to reduce anxiety levels. Patients were matched according to maternal age, number of previous pregnancies and parity, in order to reduce confounding factors.

Most of Healthy fetuses were delivered spontaneously near term of pregnancy, while maternal blood samples were made matched with FGR ones at the same gestational weeks. This explains the long time interval between sampling and delivery in Healthy. No difference in fetal blood gas analysis and Apgar score were found, because FGR were delivered before severe late deterioration happened. The difference in birth weight of the newborns is due to both the condition of growth retardation and the preterm delivery in FGR group respect to Healthy (Table 1A).

**Table 1A:** Maternal and neonatal data comparison between FGR and Healthy groups.

	**FGR (*n* = 34) (mean ± std**^†^**)**	**Health** ** (*n* = 43)** ** (mean ± std)**	*P*-value^*^
**Maternal data**			
Age (years)	33.1 ± 6.3	31.8 ± 6.7	0.423
Gravidity (*n*)	1.8 ± 1.0	1.6 ± 0.8	0.595
Parity (*n*)	0.1 ± 0.4	0.1 ± 0.4	0.851
Week of delivery	34.1 ± 2.9	36.2 ± 3.4	0.004^**^
Cesarean section (%)	100%	35%	** <0.001**
Timinig of sampling before delivery (days)	7 ± 4	38 ± 18	** <0.001**
**Neonatal data**			
Fetal pH at birth	7.27 ± 0.10	7.32 ± 0.07	0.317
pCO_2_ (mmHg)	50.35 ± 14.67	37.44 ± 3.11	0.062
pO_2_ (mmHg)	22.79 ± 11.82	25.44 ± 6.99	0.671
Base Excess (mmol/l)	−4.08 ± 3.40	−4.44 ± 7.24	0.856
Lactate (mEq/l)	4.53 ± 1.26	4.63 ± 2.58	0.935
1 min Apgar score	7.71 ± 0.91	7.37 ± 1.27	0.487
5 min Apgar score	8.47 ± 0.68	8.60 ± 0.89	0.700
Female (%)	68.9%	56.4%	**0.04**
Birth weight (g)	1592 ± 465	2544 ± 711	** <0.001**

† *STD, standard deviation*.

* *P-value for comparison between FGR and Healthy using T student two sides*.

** *Values in bold are statistically significant*.

### The Clinical Characteristics Between FGR and Healthy Showed According to the Gestational Age of FGR Subgroups Did Not Show Any Significant Difference (Table 1)

Early-FGRs were delivered before and the newborns had lower birth weight than late-FGRs, according to the different severity growth retardation in both groups. All FGRs were delivered with cesarean section and none of them in conditions of severe late deterioration, as shown by blood gas analysis data (Table 1B).

**Table 1B:** Maternal and neonatal data comparison between FGR subgroups.

	**FGR <32 weeks (mean ± std**^†^**)**	**FGR 32–37 weeks (mean ± std)**	*P*-value^*^
**Maternal data**			
Age (years)	32.9 ± 4.2	33.3 ± 8.3	0.862
Gravidity (*n*)	1.7 ± 0.9	1.8 ± 1.2	0.804
Parity (*n*)	0.1 ± 0.2	0.2 ± 0.6	0.352
Week of delivery	32.8 ± 3.1	35.7 ± 1.6	0.004^**^
Cesarean section (%)	100	100	1
Timing of sampling before delivery (days)	7 ± 4	8 ± 5	0.31
**Neonatal data**			
Fetal pH at birth	7.27 ± 0.13	7.27 ± 0.07	0.859
pCO_2_ (mmHg)	49.26 ± 17.4	51.53 ± 11.56	0.685
pO_2_ (mmHg)	24.76 ± 17.89	20.81 ± 4.18	0.728
Base Excess (mmol/l)	−4.64 ± 3.55	−3.44 ± 3.23	0.343
Lactate (mEq/l)	4.29 ± 2.63	4.65 ± 0.55	0.784
1 min Apgar score	7.06 ± 1.48	7.71 ± 0.91	0.165
5 min Apgar score	8.25 ± 0.68	8.71 ± 0.61	0.061
Female (%)	55.5	62.5	**0.04**
Birth weight (grams)	1303 ± 422	1922 ± 237	** <0.001**

† *STD, standard deviation*.

* *P-value for comparison between FGR and Healthy using T student two sides*.

** *Values in bold are statistically significant*.

Female newborns were more prevalent in FGRs than Healthy ones and in late-FGRs than early ones. A statistical analysis according to the gender of newborns was made, but no difference was found (data not shown).

All pregnant women underwent cCTG monitoring (25) to assess fetal well-being at the same gestational week and with the same frequency in all cases. In order to identify which computerized FHR parameter or parameter set is most efficient in the discrimination between non-hypoxic (Healthy) and hypoxic (FGR) fetuses, FHR was evaluated in all patients included in the experimental groups. No cCTG parameters investigated showed differences between Healthy and FGR fetuses, according to the gestational age (Table 2).

**Table 2:** Results of computerized FHR monitoring comparison between Healthy and FGR fetuses.

	**Healthy** ** (mean ± std^†^)**	**FGR** ** (mean ± std)**	*P*-value^*^
**Time parameter**			
**FCF (bpm)**			
<32nd	140.93 ± 11.88	141.97 ± 9.04	0.751
32nd−37th	143.80 ± 9.08	136.64 ± 8.71	0.070
**STV (ms)**			
<32nd	5.09 ± 1.94	5.46 ± 2.13	0.616
32nd−37th	7.10 ± 2.05	5.99 ± 2.31	0.277
**LTI (ms)**			
<32nd	18.06 ± 5.36	20.16 ± 6.20	0.320
32nd−37th	24.27 ± 5.85	20.05 ± 5.80	0.106
**Delta (ms)**			
<32nd	34.89 ± 11.72	35.99 ± 10.95	0.774
32nd−37th	44.87 ± 7.31	38.62 ± 12.00	0.225
**Interval index**			
<32nd	0.87 ± 0.07	1.01 ± 1.09	0.693
32nd−37th	0.84 ± 0.04	0.84 ± 0.04	0.863

† *STD, standard deviation*.

* *P-value for comparison between Healthy and FGR using T student two sides, according to the gestational age*.

*FHR, Fetal Heart Rate; STV, Short Term Variability; LTI, Long Term Irregularity; Delta, Delta Index*.

### miR-16-5p, miR-103-3p, and miR-27b-3p Increase in Blood of FGR Pregnant Women <32nd Week of Gestation

**Figure 1:**
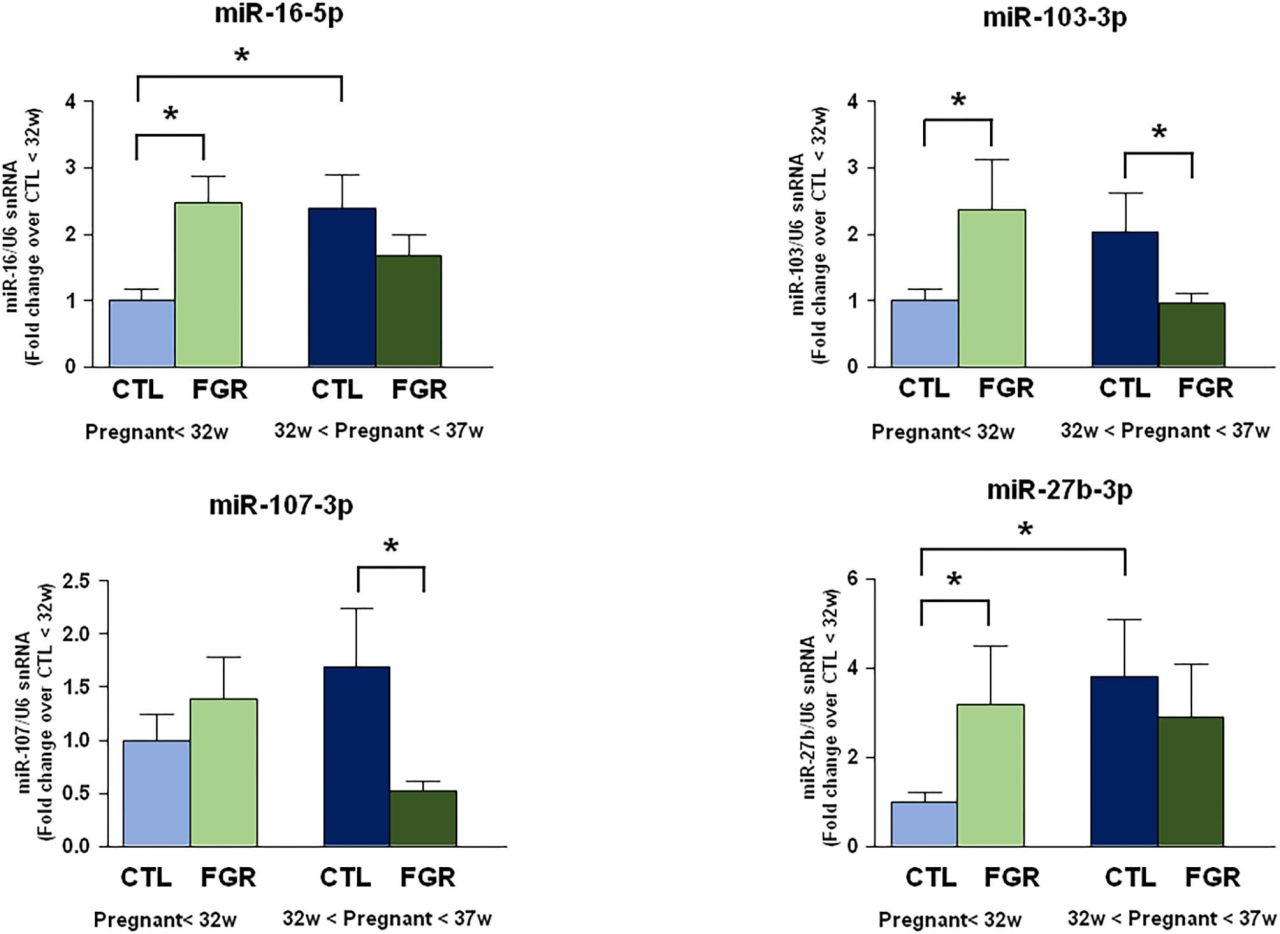
Expression analysis by real-Time PCR of hypoxia induced miRNAs in maternal blood from women with preterm FGR compared to gestation matched controls. MicroRNA levels analyzed by real-Time PCR in plasma samples are expressed as fold change of relative expression levels over the healthy group <32nd week of gestation setted at 1. Each column represents the mean ± S.E.M. Results of microRNAs expression were normalized with respect to U6 snRNA as internal control. *p < 0.05.

The expression levels of 9 microRNAs were evaluated by real-time PCR in plasma samples of pregnant women complicated by FGR before the 32nd week and between the 32nd and the 37th week of gestation, compared to control samples withdrawn at the same gestational age (Figure 1). However, only four microRNAs showed significant changes of expression. In particular, miR-16-5p, miR-103-3p, and miR-27b-3p expression levels were higher in FGR than control group before the 32nd week of gestation. On the contrary, miR-103-3p and miR-107-3p were downregulated in FGR group between the 32nd and the 37th week of gestation. Interestingly, miRNA levels showed upregulation in healthy fetuses, while they showed a progressive downregulation in fetuses with chronic hypoxia, as pregnancy progressed. The high levels of miR-16-5p, miR-103-3p, and miR-27b-3p in FGR before the 32nd week of gestation could be an early indicator of gene regulation pathways underlying the FGR development.

### Information on microRNA-Gene-Disease Ontology Interactions

The association between microRNA expression in plasma blood samples of pregnant women with FGR and predicted target genes indicate that a large group of genes may be potentially dysregulated in response to *in utero* chronic hypoxia (Table 3).

**Table 3:** A list of predicted or validated targets together with putative pathways of microRNAs dysregulated in plasma samples of pregnant women with FGR using MirPathDB 2.0.

**Candidate mirna**	**Targets**	**Evidence**	**Significant pathways involved**
hsa-miR-16-5p	A1CF (APOBEC1 complementation factor)	Predicted (int + uni)	Nitrogen compound metabolic process
	A4GNT (alpha-1,4-Nacetylglucosaminyltransferase)	Predicted (int + uni)	Nucleotide binding
	AAAS (aladin WD repeat nucleoporin)	Experimental (all)	RNA biosynthetic process
	AADAC (arylacetamide deacetylase)	Experimental (all) + predicted (int + uni)	Establishment of localization in cell
	AADAT (aminoadipate aminotransferase)	Experimental (all) + predicted (int + uni)	Nucleoplasm
hsa-miR-103a-3p family: mir-103	A1BG (alpha-1-B glycoprotein)	Predicted (int + uni)	Plasma membrane
	A1CF (APOBEC1 complementation factor)	Predicted (int + uni)	Regulation of catalytic activity
	A4GNT (alpha-1,4-Nacetylglucosaminyltransferase)	Predicted (uni)	Carbohydrate derivative binding
	AADAC (arylacetamide deacetylase)	Predicted (uni)	DNA-binding transcription factor activity
	AADAT (aminoadipate aminotransferase)	Experimental (all) + predicted (int + uni)	Nucleotide binding
hsa-miR-27b-3p	A1CF (APOBEC1 complementation factor)	Predicted (int + uni)	Nuclear chromosome
	A2ML1 (alpha-2-macroglobulin like 1)	Predicted (uni)	Cation binding
	AAGAB (alpha and gamma adaptin binding protein)	Predicted (int + uni)	Nucleotide binding
	AAK1 (AP2 associated kinase 1)	Predicted (int + uni)	Metal ion binding
	AAMDC (adipogenesis associated Mth938 domain containing)	Predicted (uni)	Endomembrane system
hsa-miR-107 family: mir-103	A1BG (alpha-1-B glycoprotein)	Predicted (int + uni)	Plasma membrane
	A1CF (APOBEC1 complementation factor)	Predicted (int + uni)	Regulation of catalytic activity
	A4GNT (alpha-1,4-Nacetylglucosaminyltransferase)	Experimental (all)	Carbohydrate derivative binding
	AADAC (arylacetamide deacetylase)	Experimental (all) + predicted (int + uni)	DNA-binding transcription factor activity
	AADAT (aminoadipate aminotransferase)	Experimental (all)	Nucleotide binding

*In the “Evidence” column of this table the acronym “int” means the intersection whereas “uni” the union of all predictions, which is a common strategy to account for putative sources of bias from target prediction tools and to balance sensitivity vs. specificity*.

Correlation analysis revealed candidate gene targets primarily involved in angiogenesis and placental development, secondly in metabolic pathways, such as cell proliferation and growth. In particular, APLN, EGFR, and FGF are targets shared by several miRNA, providing greater value to this correlation.

## Discussion

In the present pilot study, we identified for the first time a panel of hypoxia-induced miRNAs differently expressed in the maternal blood of pregnant women with FGR fetuses compared to matched age pregnancies of healthy fetuses. Our analysis involved nine microRNAs already known to be expressed in hypoxia conditions (13–19, 26, 27), five of which did not show significant expression changes in the groups assessed: miR-143-3p, let-7a-5p, miR-223-5p, miR-101a-3p, and miR-218a-5p (18, 26, 27).

By contrast, miR-16-5p, miR-103-3p, miR-107-3p, and miR-27b-3p were differentially expressed in pregnancies complicated by FGR.

In fact, miR-16-5p, miR-103-3p, and miR-27b-3p were up-regulated in FGR before the 32nd week of gestation, while miR-103-3p and miR-107-3p were down-regulated in FGR between the 32nd and 37th week of gestation. Several other studies had previously demonstrated that these four microRNAs are involved in mechanisms underlying hypoxic injury. In fact, miR-16-5p levels are correlated with severity of ischemic damage (28), and circulating miR-16-5p was also proposed as marker for the early diagnosis of ischemic stroke (20). Furthermore, studies previously performed in our laboratory showed that miR-103-3p is an epigenetic regulator of the ischemic–associated plasmamembrana protein NCX1 (29–31), and its levels correlate with ischemic injury (13). Interestingly, miR-27b-3p inhibition was demonstrated to cause both neurogenesis (15) and angiogenesis (32).

The significant up-regulation of miRNAs levels in physiologic pregnancies was opposite to the down-regulation of miRNAs levels occurring in FGR through gestation.

The target was FGR with placental insufficiency excluding growth restriction associated with preeclampsia, chromosomal and/or genetic abnormalities, and maternal pathologies. The goal was to use new methods to identify early fetuses with chronic hypoxia that require delivery before major morbidity or stillbirth occurs.

FGR condition can occur as the result of certain health problems in the mother, such as diabetes; hypertension; epilepsy; heart disease; autoimmune disorders; infections, such as rubella, cytomegalovirus, toxoplasmosis, and syphilis; kidney or lung disease; malnutrition; severe anemia, sickle cell anemia; smoking, coffee, alcohol, or drugs abusing.

Other possible fetal causes include chromosomal or genetic defects and multiple gestation. However, all maternal and fetal conditions listed were excluded from the study.

On the other hand, the term “placental insufficiency” refers to the most important pregnancy complication in which the placenta does not function adequately, because of insufficient invasion and remodeling of maternal spiral arteries that then leads to downstream effects resulting from reduced perfusion and oxidative stress, causing both FGR and preeclampsia (33).

The aspects of placental function, which are predominantly affected, determine the clinical phenotype of FGR at the time of diagnosis and progression as placental dysfunction worsens (2).

According to Baschat, the longitudinal progression of abnormal Doppler waveforms in the early FGR deterioration of utero-placental function is the following: elevated UA blood flow resistance and reduced umbilical vein flow volume precede the onset of a growth delay, followed by decreased MCA impedance and increased brain venous blood flow velocities which characterize the “brain sparing effect” (2). In late-onset FGR may present with little or no UA index elevation. Despite the seemingly “normal” placental function in the presence of a normal UA Doppler index, MCA brain sparing or a decrease in the cerebro-placental Doppler ratio (CPR) may be observed documenting decreased placental O_2_ transfer.

Therefore, to capture placental dysfunction across the phenotype of early- and late-onset FGR, inclusion of UA, MCA and Ductus venosus Doppler values are required.

Computerized FHR monitoring is the most used worldwide method to evaluate fetal well-being. In our study it did not show significant differences between Healthy and FGR fetuses according to the gestational age. Probably, major abnormalities in computerized FHR parameters occur when fetuses are affected by severe chronic hypoxia or metabolic acidosis, but all FGR enrolled in the study were delivered before these conditions developed.

In the context of the available data, we hypothesized that chronic fetal hypoxia, due to impaired uteroplacental blood flow, activates oxygen-sensitive miRNAs. By regulating target genes, these miRNAs act as triggers for the signaling cascades associated not only with responses to chronic hypoxia but also with placental development mechanisms. In placenta there are important vascular and trophoblast cell functions, impairment of these functions can result in abnormal maternal spiral arteries remodeling, placental maldevelopment and insufficiency, which in turn contributes to the etiology of FGR.

This would explain how FGR, especially early-onset, represents the final phase of an etiopathogenetic process that originates in a very early stage of pregnancy.

Probably, the evaluation of miRNA levels in maternal blood during the first trimester of pregnancy could provide the risk of FGR before it occurs.

We hope in the future to develop new screenings to assess miRNA levels in maternal blood of patients at risk for FGR in an early stage of pregnancy.

In fact, the only effective treatment for chronic fetal hypoxia is the choice of the best time to delivery before severe hypoxia occurs (8), in order to avoid the commonest causes of stillbirth, neonatal morbidity, and long-term neurological sequelae (2). However, the current clinical methods available are unable to accurately diagnose fetal hypoxia or quantify its severity. The mainstay of assessment of fetal well-being in FGR is electronic FHR monitoring. However, many authors have shown that this technique has a low positive predictive value for fetal hypoxia (1, 34).

In this study, we have identified a novel approach to non-invasively diagnose the chronic fetal hypoxia by measuring levels of circulating miRNAs in the maternal blood during pregnancy.

A non-invasive measure of fetal hypoxia would be ideal, yet we are not aware of any known maternal biomarker of fetal hypoxia. It is possible that measurement of miRNAs may therefore form the basis of a future test to determine the degree of fetal hypoxia in FGR. Several studies have investigated the association between miRNAs levels in placental tissues or in maternal blood and gestational disorders, to better understand molecular mechanisms involved in the pathophysiology of these diseases (35–37). Awamleh et al. analyzed placental tissues from two common pregnancy complications, included preeclampsia and FGR. They found 25 upregulated and 12 downregulated miRNAs in FGR samples (35). Also Hromadnikova et al. analyzed a set of miRNA from placental tissues of pregnancies complicated by FGR. They identified several miRNAs (miR-16-5p, miR-100-5p, miR-122-5p, miR-125b-5p, miR-126-3p, miR-143-3p, miR-195-5p, miR-199a-5p, miR-221-3p, miR-342-3p, and miR-574-3p) which were down-regulated in FGRs (36).

The plasma levels of several others hypoxia-induced miRNA have been recently associated with FGR (25, 37). In the last decade contrasting results have been reported on the miRNA expression levels in circulating blood and in placenta tissue. Mouillet et al. analyzed the plasma levels of a set of trophoblastic hypoxic-induced miRNA in pregnancies complicated with FGR (38). They found increased plasma levels for all miRNA examined in FGR compared to controls, but decreased levels in FGR placentas (38). By contrast, Huang et al. reported a significant increase in levels of a single miRNA (miRNA-424) in placentas of women with FGR (39). Finally, Whitehead et al. reported an upregulation in plasma levels of a set of hypoxia-regulated miRNA (miR-210, miR-424, miR-21, miR-199a, and miR-20b) of pregnancies complicated by preterm FGR compared to matched controls; moreover, miRNA plasma levels were increased according to the degree of *in-utero* hypoxia estimated (40–42).

The aforementioned scientific literature well describes different miRNA expression in mother-child pairs with adverse pregnancy outcomes respect to controls, suggesting that the evaluation of miRNA levels may serve as useful prevention and clinical tools.

However, results from several works are inconsistent and the spectrums of miRNAs observed by different studies are rather controversial (37). Such inconsistency or discrepancies can be attributed to differences in sample type, sample handling, gestational age at sampling, techniques used for miRNA profiling, and population characteristics, such as age, ethnicity, and lifestyle (37). In the present study we tried to overcome all these potential interference factors. Indeed, all pregnant women were hospitalized at our Department so that each subgroup comprised patients who had the same habits. No maternal and neonatal data differences were observed among groups.

An important aspect that should be further investigated is whether these miRNA changes are the cause or result of the FGR. In fact, if the mechanism of dysregulation involved genetic alterations at a microRNA locus, for example a deletion or a mutation affecting processing of the microRNA or amplification, it provides strong evidence for contribution of that microRNA or microRNA cluster to FGR pathogenesis. If such genetic alterations occur quite early during the multi-step process of FGR development, it would suggest a role for that microRNA in the initiation of process, providing a valuable target for therapeutic intervention.

The main limitation of our study is represented by the number of samples analyzed. However, we would like to underline that this study represents a pilot study performed to have indications on the possibility of using some miRNAs as early predictive biomarkers of FGR. On the other hand, we believe that, considering our Hospital, the numbers of patients are adequate to the cases registered. In fact, starting from a population of 2,600 pregnant women, 34 preterm FGRs, and 43 healthy fetuses fulfilled the inclusion criteria of the study. Aim of the study was to evaluate miRNA expression in the blood of Caucasian women with single pregnancy complicated by FGR not associated with other maternal (such as hypertension, preeclampsia, diabetes, autoimmune diseases, renal or respiratory failure, hemoglobinopathies, isoimmunization, alcohol, or drugs abuse) or fetal diseases (such as malformations, known genetic or chromosomal abnormalities). We also excluded cases for which all pregnancy and delivery data were not available (i.e., certain pregnancy date, cCTG with a signal loss <15%, cCTG traces with the same recording frequency, maternal blood samples collected after 2 weeks of hospitalization and at least 48 h before delivery, as well as all delivery and newborn data). This strict selection made the sample small.

Overall, from our results it is conceivable that measurement of miRNAs in maternal blood may form the basis for a future diagnostic test to determine the degree of fetal hypoxia in FGR, thus allowing the initiation of appropriate therapeutic strategies in order to alleviate the burden of this disease. In addition, the identification of potential targets for the selected miRNAs will pave the way for the development of innovative pharmacological strategies exploiting these druggable targets.

## Data Availability Statement

The datasets presented in this study can be found in online repositories. The names of the repository/repositories and accession number(s) can be found in the article/supplementary material.

## Ethics Statement

The studies involving human participants were reviewed and approved by Azienda Ospedaliera Universitaria Federico II, Naples, Italy. The patients/participants provided their written informed consent to participate in this study.

## Author Contributions

GP, LA, FZ, and GM contributed to the conception and design of the study. ST, PC, and MC contributed to the acquisition and analysis of data. ST, PC, AV, GP, LA, and GM contributed to drafting a significant portion of the manuscript, tables, and figures. ST examined and collected all the clinical data. PC evaluated the expression of miRNAs in blood samples. All authors contributed to the article and approved the submitted version.

## Conflict of Interest

The authors declare that the research was conducted in the absence of any commercial or financial relationships that could be construed as a potential conflict of interest.
